# Toxicity of Common Acaricides, Disinfectants, and Natural Compounds against Eggs of *Rhipicephalus annulatus*

**DOI:** 10.3390/pathogens13100824

**Published:** 2024-09-24

**Authors:** Samar M. Ibrahium, Abdel-Azeem S. Abdel-Baki, Sahar M. Gadelhaq, Shawky M. Aboelhadid, Hesham A. Mahran, Saleh Al-Quraishy, Abdulrahman Reyad, Asmaa A. Kamel

**Affiliations:** 1 Parasitology Department, Animal Health Research Institute (AHRI), Agriculture Research Center (ARC), Fayoum 16101, Egypt; drsamarmahmoud333@yahoo.com; 2 Department of Parasitology, Zoology Department, Faculty of Science, Beni-Suef University, Beni-Suef 62511, Egypt; aabdelbaki@science.bsu.edu.eg; 3 Parasitology Department, Faculty of Veterinary Medicine, Minia University, Minia 61519, Egypt; sahargad25@gmail.com; 4 Parasitology Department, Faculty of Veterinary Medicine, Beni-Suef University, Beni-Suef 62511, Egypt; asmaa.abdelaal@vet.bsu.edu.eg; 5 Hygeine Department, Faculty of Veterinary Medicine, Beni-Suef University, Beni-Suef 52611, Egypt; hygienemail@gmail.com; 6 Zoology Department, College of Science, King Saud University, Riyadh P.O. Box 2455, Saudi Arabia; squraishy@ksu.edu.sa; 7 Hydrobiology Department, Debrecen University, 4002 Debrecen, Hungary; ragabb700@mailbox.unideb.hu

**Keywords:** cattle tick, ovicide, acaricides, disinfectants, carvacrol, thymol

## Abstract

Ticks pose a significant threat due to their ability to lay thousands of eggs, which can persist in the environment for extended periods. While the impact of various compounds on adult and larval ticks has been studied, research on their efficacy against tick eggs is limited. This study evaluated the ovicidal activity of commercial acaricides, disinfectants, and natural products against *Rhipicephalus annulatus* eggs using the egg hatch assay (EHA). Deltamethrin and cypermethrin caused a non-significant inhibition of hatching (IH%), even at concentrations higher than the recommended levels. By contrast, the acaricides chlorpyrifos, phoxim, and amitraz significantly inhibited hatching at all tested concentrations. Ivermectin also demonstrated significant IH% at various concentrations but did not fully inhibit the hatching process. Among the disinfectants tested, Virkon-S^®^, TH4, and Chlorox showed insignificant effects, whereas formalin achieved an IH% of only 34.1% at a high concentration of 200 mg/mL. Natural products, carvacrol and thymol, exhibited significant ovicidal activity, with a significant IH%. In a semi-field application, phoxim (0.5 mg/mL) and deltamethrin (0.05 mg/mL) were sprayed on tick eggs on pasture soil from a farm. The results indicated that phoxim-treated eggs had a 40% IH%, while deltamethrin-treated eggs showed only an 8.79% IH%. In conclusion, the acaricides amitraz, phoxim, and chlorpyrifos, as well as the natural products carvacrol and thymol, caused significant toxicity to *R. annulatus* eggs.

## 1. Introduction

*Rhipicephalus (Boophilus) annulatus* (Say, 1821) is prevalent in tropical and subtropical regions, including Mexico, the Middle East, Africa, South America, and Europe [1,2]. Heavy infestations of this tick species can significantly reduce animal productivity, cause anemia, and damage hides. Additionally, *R. annulatus* serves as a vector for diseases such as anaplasmosis (*Anaplasma marginale*) (Theiler, 1910) [3,4] and lumpy skin disease virus [5]. According to de Castro et al. [6], 1.3 billion cattle worldwide are at risk of tick-borne diseases (TBPs), with an estimated annual economic impact of USD 22 to 30 billion. In 2020, this economic burden was the highest it had been in over two decades [7].

*R. annulatus* is a one-host tick that completes its life cycle in 3–4 weeks, leading to a substantial tick burden [8]. The adult female lays eggs that hatch into larvae, which attach to a host (cattle in the case of *R. annulatus*), feed, and then molt into nymphs that mature into adults—all within the same host. Female *R. annulatus* typically deposit eggs in crevices, debris, or under stones [9]. On average, a female tick produces egg masses weighing 29.5 ± 9.03 mg, containing about 1455.3 ± 434.5 eggs, with a hatching rate of 83.5 ± 2.94% [9]. The pre-oviposition, oviposition, and pre-hatching periods for females average 4.1 ± 1.3 days, 8.6 ± 0.85 days, and 21.03 ± 2.99 days, respectively, meaning eggs can remain on the ground for at least a month [8]. Jagannath et al. [10] reported that in southern India, the preoviposition period for *R. annulatus* ranged from 6 to 7 days at temperatures between 26 and 40 °C. The oviposition period lasted from 11 to 16 days, with each female laying between 1548 and 5837 eggs. The most egg production occurred between 1 and 3 days after the start of oviposition. Additionally, Davey [11] noted that each female laid approximately 2700 eggs at temperatures between 25 and 27 °C. Despite being exposed to environmental conditions that might promote microbial attacks, tick eggs remain viable without signs of bacterial colonization [12,13]. This is due to the Géné’s organ in ticks, which secretes a wax coating on the eggs that prevents desiccation and protects against microbial invasion. The wax is a complex mixture of long-chain alkanes and fatty acid esters [12,13,14,15].

Off-host, engorged females, larvae, and eggs are subject to environmental challenges, including extreme temperatures, humidity fluctuations, water and energy loss, and starvation [16]. Temperature and humidity are key ecological factors that affect the survival and questing activity of a tick’s off-host stages [17]. The incubation period for *R. annulatus* eggs, for example, ranges from 52 days at 20 °C to 16 days at 35 °C. [18]. Temperatures between 20 and 35 °C and relative humidity levels above 70% are considered optimal for the survival of engorged females and eggs. The duration of both the preoviposition and egg incubation periods decreases as temperatures increase [19,20,21].

There has been limited research on the efficacy of acaricides against tick eggs. Haque et al. [22] reported that flumethrin and amitraz completely inhibited hatching, while only high concentrations of cypermethrin, deltamethrin, and fenvalerate significantly affected the hatching of *Rhipicephalus (Boophilus) microplus* (Guglielmone, 2010) eggs. Given that eggs can survive in the environment for at least a month, it is crucial to include them in tick control strategies. Sharma et al. [23] evaluated the ovicidal effectiveness of cypermethrin and amitraz on *R. microplus* eggs, finding that both compounds completely prevented egg hatching in their assays. By contrast, deltamethrin did not fully inhibit egg hatching. Additionally, Prado-Ochoa et al. [24] reported that carbamates were able to inhibit up to 100% of egg hatching in *R. microplus*.

In Egypt, several chemical acaricides are approved for tick control. The most commonly used acaricides are deltamethrin (5%) and cypermethrin (6%) [25]. Other acaricides in use include chlorpyrifos (25%), phoxim (50%), and amitraz (20%) [26]. Additionally, ivermectin is widely used for controlling internal nematodes, as well as external mites and ticks [27]. Natural compounds such as carvacrol and thymol also exhibit significant acaricidal activity. Carvacrol has been shown to cause significant mortality in various stages of *R. annulatus* and *R. sanguineus* [28], while thymol has demonstrated potent activity against different stages of these tick species as well [29,30]. Common disinfectants such as Virkon-S, TH4, and Chlorox are widely used in animal farms to address various infections [31,32]. The timing of female oviposition should be considered, as it can be advantageous to implement control measures that target eggs, potentially rendering them non-viable [12,33]. The chemical composition of the secretion synthesized and secreted by *R. sanguineus* Gené’s organ cells, in addition to the strong lipid positivity, would respond weakly to polysaccharide components and calcium [34]. Thus, the goal of this study was to assess the ovicidal activity of conventional acaricides (deltamethrin, cypermethrin, chlorpyrifos, phoxim, amitraz and ivermectin), disinfectants (Virkon-s, Chlorox, formaline), and natural products (carvacrol and thymol) against *R. annulatus* eggs.

## 2. Materials and Methods

### 2.1. Acaricides, Chemicals, and Natural Products

Commercial acaricides, Alphatac (6.0% EC cypermethrin) and Super Butox (5.0% EC deltamethrin), Amitraz (20.0%), chlorpyrifos (25.0%), phoxim (50.0%), and Ivermectin (99.0% pure powder) were purchased from Khafer Elzayat Pesticides and Chemicals, Khafar El-zayat, Egypt. Disinfectants such as Virkon S, TH4, Chlorox, and formalin were purchased from a local company. The natural compounds carvacrol (100.0%) and thymol crystal (99.0%) were acquired from Loba Ind. Company, Mumbai, India. Ethyl alcohol and Triton X-100 were obtained from BioChem, Giza, Egypt.

### 2.2. Preparation of Different Common Acaricides

Acaricides were dissolved in distilled water to create a range of concentrations for experimental analysis. The base concentration, denoted as “X,” corresponded to the manufacturer’s recommended dosage (0.5 mL/L). To assess the efficacy of different concentrations, solutions were prepared at 1/2 X, X, 2 X, 4 X, and 8 X multiples of the base concentration. The specific concentrations used for each acaricide were determined based on its active ingredient composition.

### 2.3. Ivermectin Preparation

A stock solution of ivermectin (1% concentration) was initially prepared using 100% ethanol as a solvent [35]. To enhance solubility and facilitate subsequent dilutions, a 1% ethanol solution containing 2% Triton X was created. This solution served as the basis for preparing a series of ivermectin dilutions ranging from 5 mg/mL to 0.09 mg/mL.

### 2.4. Disinfectants Preparations

Disinfectant solutions were prepared at varying concentrations using distilled water as a diluent. Virkon-S^®^ and TH4 were diluted to final concentrations of 5, 10, and 20 mg/mL. Commercial Chlorox (5%) was diluted to yield solutions of 1, 2, and 4 mg/mL. Formalin (40%) was diluted to produce solutions of 50, 100, and 200 mg/mL.

### 2.5. Natural Compounds Preparation

Pure carvacrol and thymol were dissolved in absolute ethanol to create stock solutions [36]. A series of dilutions were prepared using 70% ethyl alcohol to achieve concentrations of 100, 50, 25, 12.5, 6.25, 3.12, and 1.56 mg/mL.

### 2.6. Eggs Collection of R. annulatus

Live, engorged adult female *R. annulatus* ticks were collected from cattle in private farms in the Beni-Suef (29°04′ N 31°05′ E) and Fayoum (29.308374° N 30.844105° E) provinces over a six-month period from March to September. These farms had a history of tick infestations without recent acaricide treatments. Collected ticks were transported in plastic vials to the Parasitology department at Beni-Suef University. After cleaning the ticks with water to remove any debris or hair from the infested animals and drying them carefully with filter paper, the ticks were labeled and then incubated for two weeks in a BOD incubator to initiate egg laying. The eggs were collected 10–12 days following deposition, as one-day-old eggs were more susceptible to acaricides than eggs aged 10–12 days [37]. The eggs were retrieved after 10–12 days of incubation and blended with a small plastic spatula to ensure homogeneity. Subsequently, the collected eggs were divided into 100 mg portions and placed into separate glass tubes. 

### 2.7. Egg Hatch Assay (EHA)

An egg hatching assay was conducted following the methodology described by Ribeiro et al. [38]. The egg hatching assay was conducted using 100 mg of eggs, which is approximately 1000 eggs [8]. Each treatment was applied to a total of 5000 eggs, based on 5 replicates, with each replicate containing 1000 eggs. One hundred milligrams of eggs (5000 eggs) were submerged in 1 mL of a test solution in glass test tubes for two minutes at ambient temperature (approximately 27 °C). The tubes were turned upside down to remove excess solution into a cotton plug, and the treated eggs were incubated in a biological oxygen demand (BOD) incubator at 28 ± 1 °C and 85 ± 5% relative humidity for 21 days to facilitate hatching. The hatched larvae were examined under a microscope to assess their movement and viability. Control groups, including distilled water, 70% ethyl alcohol, and 1% Triton X, were subjected to the same protocol. Each treatment group consisted of five replicates. The following parameters were compared:

(1) $$A.\;Hatching\;(\%)=\frac{The\;number\;of\;hatched\;larvae}{The\;total\;number\;of\;incubated\;eggs}$$

(2) $$B.\;Percentage\;inhibition\;of\;hatching\left(IH\%\right)=\;100\;\times \frac{Hatching\;\%\;control-Hatching\;\%\;treated}{Hatching\;\%\;control}$$

### 2.8. Extended In Vitro Inhibition of Egg Hatching Bioassay

A soil-based experimental model was established to assess the ovicidal efficacy of treatments under semi-field conditions. Approximately 10 g of pasture soil (pH 7.5) was uniformly distributed within 9 cm diameter Petri dishes. Subsequently, 100 mg of tick eggs were centrally placed on the soil surface. Treatment solutions, including deltamethrin (0.05 mg/mL) and phoxim (0.5 mg/mL) as test compounds and distilled water as a control, were applied to the soil–egg mixture. Petri dishes were then incubated under controlled conditions (28 ± 1 °C, 85 ± 5% relative humidity) for 14–28 days to allow for egg hatching. Each treatment group consisted of five replicates.

### 2.9. Statistical Analysis

To determine significant differences in egg-hatching rates among treatment groups, a one-way ANOVA was conducted, followed by Duncan’s multiple range test for pairwise comparisons. The level of significance was set at *p* < 0.05. To assess the toxicity of the tested compounds, lethal concentrations (LC_50_ and LC_90_) and their corresponding 95% confidence intervals were calculated using Probit analysis [39]. All statistical analysis was conducted using IBM SPSS for Windows, version 22 (IBM, Armonk, NY, USA).

## 3. Results

### 3.1. Ovicidal Activity of Acaricides

**Figure 1:**
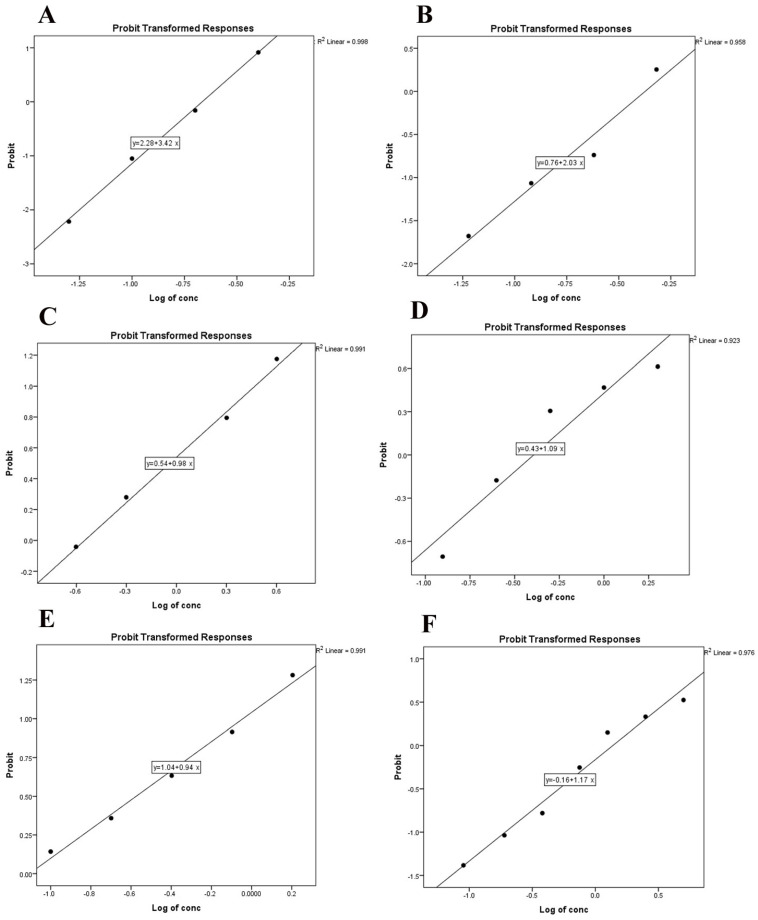
Dose–probit hatchability curve of treated against eggs of *R. annulatus*; (**A**) deltamethrin-treated eggs, (**B**) cypermethrin-treated eggs, (**C**) cholorpyrifos-treated eggs, (**D**) phoxim-treated eggs, (**E**) amitraz-treated eggs, and (**F**) ivermectin-treated eggs.

Lower concentrations of deltamethrin and cypermethrin (0.5 X and X) exhibited negligible effects on egg hatching. However, a marked increase in efficacy was observed at the highest concentration (8 X), resulting in inhibition rates of 82.0% and 60.0%, respectively, for deltamethrin and cypermethrin (Figure 1A,B). By contrast, chlorpyrifos, phoxim, and amitraz demonstrated significant ovicidal activity even at low concentrations (0.5 X), with inhibition rates increasing proportionally with dosage (Table 1). Amitraz and phoxim exhibited the most potent ovicidal effects, achieving inhibition rates of 90.0% and 88.0%, respectively, at the highest concentration (8 X) (Figure 1C–E). Larvae hatched from eggs treated with 4 X concentrations of amitraz, phoxim, and chlorpyrifos exhibited weak and sluggish movements (Figure S1). When comparing the concentration per percent inhibition of hatching in *R. annulatus* eggs, amitraz reached the LC_50_ at the lowest concentration of 0.077 mg/mL, while cypermethrin required the highest concentration of 0.408 mg/mL to achieve the same effect, based on the commercial solutions used (Table 1).

### 3.2. Ovicidal Activity of Ivermectin

**Figure 2:**
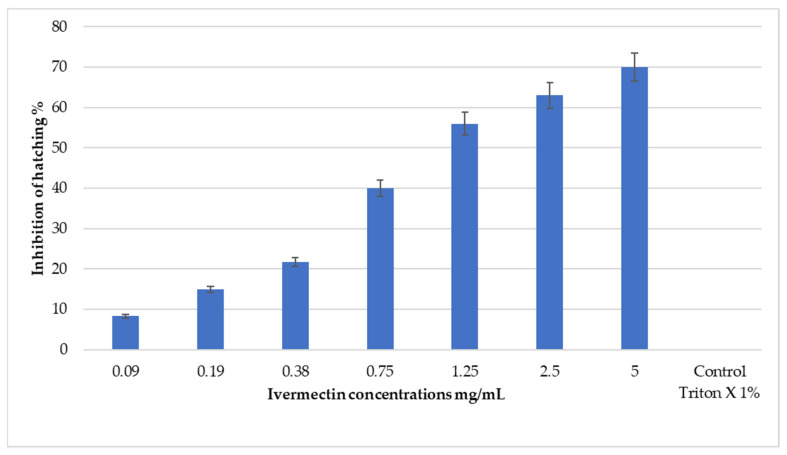
Percentage inhibition of hatching (IH%) of *R. annulatus* eggs treated by different concentrations of ivermectin.

Various concentrations of ivermectin were tested due to the absence of a recommended dosage specifically for tick eggs. Although none of the concentrations achieved 100% inhibition of hatching (IH%), higher concentrations (>0.75 mg/mL) showed significant IH% exceeding 50%. By contrast, lower concentrations (≤0.38 mg/mL) resulted in an IH% of 20% or less (Figure 1F and Figure 2). Additionally, the LC_50_ and LC_90_ values for ivermectin were determined to be 1.25 mg/mL and 15.8 mg/mL, respectively (Table 1, Figure S2).

### 3.3. Ovicidal Effect of Disinfectants

Virkon-S^®^ and TH4, commonly used disinfectants, exhibited a low inhibition of hatching percentage (IH%) against tick eggs. The IH% remained low, ranging from 18.0% to 23.0%, even when the concentration was increased fourfold, from 5 to 20 mg/mL (Table 2). Similarly, Chlorox showed a limited effect on egg hatching (Table 2). By contrast, formalin at a concentration of 200 mg/mL resulted in an IH% of 34.1% in tick eggs, while lower concentrations demonstrated only minimal IH% (Table 2, Figure S3).

### 3.4. Ovicidal Effect of Selected Natural Products

**Figure 3:**
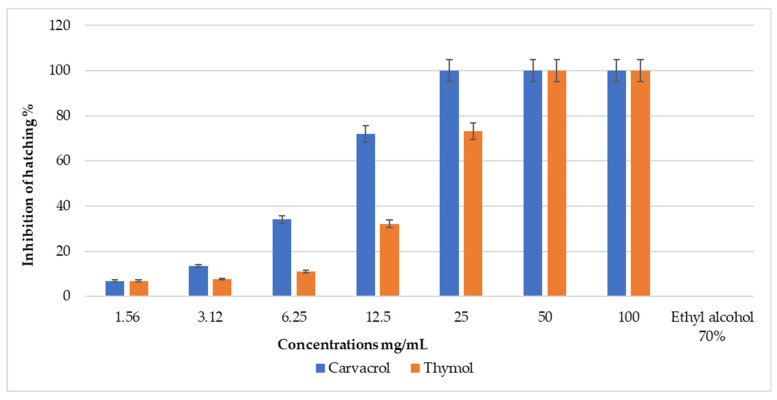
Percentage inhibition of hatching (IH%) of *R. annulatus* eggs treated by natural products of plant origin.

**Figure 4:**
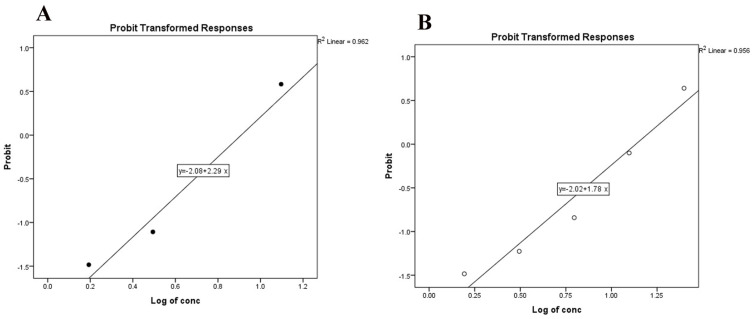
Dose–probit hatchability curve of treated against eggs of *R. annulatus*; (**A**) carvacrol-treated eggs and (**B**) thymol-treated eggs.

The natural compounds thymol and carvacrol were highly effective in inhibiting egg hatching, achieving 100% inhibition (100% IH) at higher concentrations of 100 and 50 mg/mL for both compounds, and at 25 mg/mL for carvacrol. However, at lower concentrations, the IH% of eggs was significantly reduced (Figure 3 and Figure 4). The LC_50_ concentrations for carvacrol and thymol were determined to be 9.1 mg/mL and 18.0 mg/mL, respectively (Table 1, Figure S4). The LC_90_ for carvacrol and thymol were found to be 15.4 mg/mL and 30.7 mg/mL, respectively (Table 1).

### 3.5. Extended In Vitro Inhibition of Egg Hatching Bioassay Results Using Soil

The IH% for eggs treated with deltamethrin at a concentration of 0.05 mg/mL was 8%, while eggs treated with phoxim at a concentration of 0.5 mg/mL (X) had a significantly higher IH% of 40%. The larvae that hatched from eggs treated with deltamethrin were generally viable, whereas the larvae from eggs treated with phoxim were either dead or exhibited weak movements (Table 3).

## 4. Discussion

Controlling tick populations is a complex task influenced by various factors. Traditionally, direct acaricide application to animals has been the primary method, but its effectiveness is often short-lived due to the tick’s life cycle [40]. Tick populations fluctuate based on environmental conditions like climate and vegetation, which significantly impact the off-host stages [41]. The life cycle of ticks underscores the importance of a comprehensive approach. While treating animals to eliminate adult ticks is essential, targeting the off-host stages, particularly eggs, is equally critical. By interrupting the reproductive cycle, it is possible to reduce future tick populations [42]. To achieve long-term tick control, integrating various strategies is necessary. This includes the judicious use of acaricides, habitat modification to reduce tick populations, and public awareness campaigns to promote preventive measures. Additionally, exploring alternative control methods holds promise for the future [16,17].

The current investigation revealed that deltamethrin and cypermethrin were ineffective at inhibiting egg hatching at low concentrations (0.5 X and X). However, at the highest concentration tested—8 times the recommended level (8 X)—significant inhibition of egg hatching was observed. The limited efficacy of deltamethrin and cypermethrin at standard concentrations is likely due to resistance in local tick isolates in the Beni-Suef region of Egypt. This conclusion aligns with previous studies on acaricide resistance in *R. annulatus* from this area (Beni-Suef, Egypt) [36,43]. Tick resistance is genetically inherited and passed on to offspring, which likely explains why the eggs also exhibited resistance to these acaricides. This is further supported by the findings of Arafa et al. [36] who identified a C190A single-point mutation in the Na-channel gene of deltamethrin-resistant *R. annulatus* in Egypt using PCR-HRM analysis. Notably, eggs exposed to deltamethrin showed lower hatchability compared to those exposed to diazinon [44]. While studies from India and Egypt by Ravindran et al. [45] and Khalaf-Allah [46], respectively, reported high efficacy of cypermethrin against adult *R. annulatus*. Other research by Rodrguez-Hidalgo et al. [47] in Ecuador and Sousa et al. [48] in Brazil documented cypermethrin resistance in *R. microplus* (Guglielmone, 2010) populations. Interestingly, Ravindran et al. [49] also found that deltamethrin completely inhibited *R. annulatus* egg hatching at 30 ppm, highlighting regional variations in resistance patterns.

Chlorpyrifos, phoxim, and amitraz demonstrated significant inhibition of egg hatching (IH%) even at concentrations lower than the recommended levels, with the maximum IH% achieved at the highest concentration (8 X). Among these, amitraz proved to be the most effective acaricide, with an IH% of 90.0%. The effectiveness of phoxim aligns with the findings of Abdel Aziz et al. [26] who reported that phoxim significantly inhibited the hatching of *R. sanguineus* (Latreille, 1806) eggs. Amitraz, a triazapentadiene compound from the formamidine pesticide family, has long been used in veterinary medicine [50]. Several studies have shown that amitraz, at various concentrations (200, 250, 300, and 350 ppm), significantly inhibited the hatchability of *R. annulatus* eggs when compared to the control [51]. Amitraz works by reducing glutamate release in susceptible ticks, maintaining the Mg^+2^ block, which further restricts Ca^+2^ entry through NMDA receptors, leading to continuous excitation and paralysis [52]. At a concentration of 300 ppm, which is commonly used in field conditions, amitraz inhibited fecundity by 96.74% and completely blocked egg eclosion [49]. Coumaphos, diazinon, dioxathion, and chlorpyrifos were found to be most effective in suppressing the hatching of 1-day-old eggs but showed no effect on eggs that were 10 or 20 days old [37].

Regarding the mode of action, phoxim, an organophosphate, inhibits acetylcholinesterase at the synaptic junction, leading to the accumulation of acetylcholine and extending its muscarinic and nicotinic effects in the central nervous system, autonomic nervous system, and neuromuscular junctions [53]. On the other hand, deltamethrin, a pyrethroid, prolongs neuronal depolarization by interacting with sodium channels [54]. The effectiveness of both acaricides in suppressing egg hatching can be attributed to their ability to penetrate the eggshells and exert their effects on the larvae within the eggs through the same mechanisms of action they use against larvae and adults [55].

Ivermectin demonstrated significant inhibition of egg hatching (IH%) but required a high concentration, with an LC_50_ of 1.25 mg/mL. This finding aligns with the study by El-Bahy et al. [44], which reported a 71.6% in vitro efficacy of ivermectin on the hatchability of *R. annulatus* eggs. Ivermectin is a macrocyclic lactone (ML) widely used as an acaricide for controlling tick populations [56]. The pharmacokinetics of MLs, like ivermectin, in arthropods is well documented, primarily due to their strong affinity for glutamate-gated chloride channels (Glu-Cl) in muscle and nerve tissues. The inhibition of these channels leads to a gradual and irreversible increase in membrane conductance, causing somatic muscle paralysis and ultimately the death of the parasite [57].

Common disinfectants like Virkon-S, TH4, and Chlorox exhibit a limited inhibition of hatchability (IH%) against tick eggs, even when applied at concentrations higher than the recommended rates. These disinfectants are widely used in animal farms to combat various infections [31,58]. Virkon^®^ S, as noted by Gasparini et al. [59], works by oxidizing sulfur bonds in proteins and enzymes, disrupting cell membrane function, and rupturing the cell wall. Chlorine, a potent oxidizing agent, similarly disrupts protein cellular function [60]. Formalin, at a concentration of 200 mg/mL, achieved an IH% of 34.1% in tick eggs, even at relatively low concentrations. Formaldehyde is known to interact with proteins, DNA, and RNA [61], and has been proven effective as an oocysticide [32] and as a bactericidal, sporicidal, and virucidal agent [62,63]. The limited effectiveness of these disinfectants against tick eggs could be due to the wax coating that protects the eggs from external chemicals [12]. The effectiveness of disinfectants is also influenced by several factors, including the type of disinfectant, application method, exposure time, natural microbial population, surface material, and environmental conditions like temperature. The efficacy of most disinfectants decreases under low temperatures, short contact times, and on porous surfaces. Therefore, when applying disinfectants in field situations, it is crucial to consider the environmental temperature, duration of disinfection, and the nature of the target surface to ensure successful outcomes [31,58].

Carvacrol and thymol are emerging as promising acaricides, particularly due to their significant ovicidal activity, with carvacrol demonstrating superior effectiveness compared to thymol. While the acaricidal properties of these natural products have been widely documented, their ovicidal potential has not been as extensively studied. Carvacrol has shown acaricidal efficacy against various tick species, including *Rhipicephalus microplus* (Canestrini, 1888), *Amblyomma americanum* (Koch, 1844), *Hyalomma marginatum* (Koch, 1844), *Rhipicephalus turanicus* (Pomerantzev, 1940), and *Rhipicephalus sanguineus* [64,65,66]. The ovicidal effects of thymol have been supported by studies such as those conducted by Arafa et al. [36] and Tabari et al. [67]. However, contrasting results have been observed, as De Oliveira Monteiro et al. [68] reported that thymol at a 2% concentration had no effect on R. microplus eggs. This discrepancy highlights the need for further research into the ovicidal properties of these compounds to better understand their potential in tick control strategies.

The effectiveness of ovicidal compounds can be linked to their interaction with egg wax, a complex mixture of long-chain alkanes and fatty acid esters that serves as a protective barrier for tick eggs [12]. Research by Xavier et al. [69] into lipid metabolism highlighted the role of Gené’s organ in synthesizing, modifying, and oxidizing fatty acids. Their study identified critical proteins and pathways involved in the secretion of egg wax and its role in egg development. Targeting these components and pathways could provide innovative strategies for tick control by decreasing egg viability in the environment. Effective acaricides should be able to dissolve or disrupt this wax layer [34]. Both carvacrol and thymol, among other tested acaricides, have shown the capability to perform this function, suggesting their potential as effective ovicidal agents.

This study focused on tick eggs derived from isolates resistant to deltamethrin and cypermethrin, limiting the availability of susceptible samples. This reflects a broader challenge in the study region, where resistance to common acaricides is prevalent [36,43,56]. Consequently, a substantial portion of our research has been dedicated to developing strategies to address this critical issue.

Another key element is the feasibility of applying ovicidal products to the ground and the impact on non-target organisms. Pesticides, including insecticides, can contaminate soil, water, and vegetation. This contamination can harm non-target organisms such as birds, fish, and beneficial insects [70]. Heavy pesticide treatment can lead to a decline in beneficial soil microorganisms, reducing nutrient retention in the soil and negatively impacting long-term soil health [71]. Pesticides can drift or volatilize, contaminating air, soil, and non-target vegetation, leading to broader environmental impacts [70].

To mitigate these risks, it is essential to take precautions when applying insecticides. First, the timing of application is critical; it should coincide with the most vulnerable stage of the pest’s life cycle to maximize effectiveness while minimizing harm to non-target organisms. Favorable weather conditions that reduce the potential for drift or volatilization should also be chosen to protect surrounding areas [72]. Insecticides with high efficacy and low environmental impact should be prioritized. Organophosphate-based products, developed as alternatives to more toxic chemicals, are one option due to their broad action and application in both agriculture and sanitation [73]. However, their use still requires caution due to their potential toxicity [73]. Alternatively, synthetic pyrethroids, derived from natural pyrethrins found in *Chrysanthemum cinerariaefolium*, have emerged as less toxic and more biodegradable alternatives to DDT and other insecticides [74,75].

In the context of tick control, particularly for one-host ticks like *R. annulatus*, which complete their life cycle in a single environment, the application of pyrethroids should focus on areas where ticks are most likely to lay eggs. Targeting specific sites, such as animal shelters and crevices in shelter walls, can help effectively manage tick populations while minimizing environmental impact [16,17]. In terms of compound safety, amitraz is generally considered safe when used as recommended for flea control in dogs, and it has been widely used as an alternative in rotating management techniques for treating pyrethroid-resistant ticks [76,77,78]. Phoxim^®^, a highly powerful and adaptable organophosphate pesticide used in veterinary and agricultural settings, is also safe when administered as directed. The World Health Organization (WHO) has defined safe dose limits (0–4 µg/kg body weight or 0.1% aqueous solution) to ensure that Phoxim^®^ can be used efficiently without impacting the health of treated animals [79,80]. Ivermectin’s impact on global health is undeniable. Its inclusion alongside penicillin and aspirin as a “wonder drug” is a testament to its remarkable efficacy and safety [81]. By targeting a wide range of parasitic diseases that disproportionately affect impoverished populations, ivermectin has significantly improved the lives of millions worldwide [82]. This drug’s versatility and its role in combating neglected tropical diseases solidify its status as a cornerstone of modern medicine [83]. Also, carvacrol and thymol are the primary constituents of essential oils derived from the Lamiaceae plant family [84]. Historically employed in traditional medicine, these compounds have demonstrated expectorant, anti-inflammatory, antiviral, antibacterial, and antiseptic properties. Their safety profile has led to widespread acceptance for various applications [85,86,87].

In conclusion, to obtain a considerable ovicide impact (over 50% IH), amitraz, phoxim, and chlorpyrifos may be used at the recommended concentrations. Additionally, carvacrol functions effectively and has the potential to kill eggs at a concentration of 18 mg/mL. It is noteworthy that even at high doses, ivermectin can have ovicide effects; nonetheless, this is not advised. Furthermore, typical disinfectants only partially affect tick eggs.

## Figures and Tables

**Table 1:** Dose-percent inhibition of *R. annulatus* eggs treated by various acaricides natural products of plant origin.

Acaricide	LC_50_ (95% CL) mg/mL	LC_90_ (95% CL) mg/mL	Χ^2^	df	Slope ± SE	*p* *	*R* ^2^
Deltamethrin	0.216 (0.195–0.242)	0.520 (0.436–0.658)	0.526	3	3.36 ± 0.29	0.913	0.998
Alpha-cypermethrin	0.408 (0.342–0.514)	1.544 (1.079–2.617)	5.008	3	2.22 ± 0.23	0.171	0.958
Cholopyrifos	0.398 (0.303–0.506)	5.96 (3.44–14.59)	5.177	3	1.09 ± 0.14	0.159	0.923
Phoxim	0.279 (0.156–0.401)	5.88 (3.54–14.27)	0.392	3	0.97 ± 0.15	0.942	0.991
Amitraz	0.077 (0.035–0.120)	1.86 (1.14–4.53)	0.336	3	0.93 ± 0.15	0.953	0.991
Ivermectin	1.25 (1.02–1.57)	15.8 (10.02–29.40)	9.655	5	1.18 ± 0.09	0.086	0.976
Carvacrol	9.10 (8.21–9.97)	15.4 (14.37–17.99)	1.32	5	2.02 ± 0.09	0.933	0.990
Thymol	18.0 (16.49–19.97)	30.7 (28.70–35.62)	0.997	5	1.01 ± 0.04	0.963	0.993

CL, confidential limit; *X*^2^, Chi-square; LC, lethal concentration; df, degree of freedom, * *p* > 0.05 is non-significant.

**Table 2:** Percentage inhibition of hatching (IH%) of *R. annulatus* eggs treated by common disinfectants.

Disinfectants/Conc	Percentage Inhibition of Hatching (IH%) ± SE		
	5 mg/mL	10 mg/mL	20 mg/mL
Virkon^®^ S	7.66 ± 2.89	7.28 ± 2.39	9.95 ± 1.52 *
TH4	6.89 ± 2.29	8.81 ± 3.51	11.1 ± 2.39 *
	1 mg/mL	2 mg/mL	4 mg/mL
Chlorox (5%)	6.89 ± 3.04	7.28 ± 1.75	8.81 ± 1.75
	50 mg/mL	100 mg/mL	200 mg/mL
Formalin	6.51 ± 1.75	6.89 ± 2.29	34.1 ± 2.89 *
Control group Distilled water	0.00 ± 0.00	0.00 ± 0.00	0.00 ± 0.00

(*) significant for DW, *p* < 0.05.

**Table 3:** Semi-field application of deltamethrin and phoxim acaricides at the rate of the recommended concentration.

Treatments	Phoxim (0.5 mg/mL) Treated Eggs ± SE	Deltamethrin (0.05 mg/mL) Treated Eggs ± SE	Untreated Eggs DW Control
Percentage inhibition of hatching (IH%)	40.0 ± 5.08 *	8.79 ± 4.06	0.00 ± 0.00
Viability of hatched larvae	Slow motion or dead	Viable	Viable

(*) significant for DW (distilled water), *p* < 0.05.

## Data Availability

All related data to this work are available in this manuscript.

## Supplementary Materials

The following supporting information can be downloaded at: https://www.mdpi.com/article/10.3390/pathogens13100824/s1, Figure S1: A. Deltamethrin treated eggs at concentration 8X with 82% percent of inhibition of hatching (IH%), with eggs collapsed and destructed eggs. B. Cypermethrin treated eggs at concentration 2X with IH% = 14.33% and large number of viable larvae. C. Chlorpyrifos treated eggs at concentration 1/2X with IH% = 16.3% and large number of viable larvae. D. Amitraz treated eggs at concentration X with IH% = 64% and shells of hatched eggs and larvae. E. Phoxim treated eggs at concentration X with IH% = 61.3% and shell of hatched eggs and larvae; Figure S2: Ivermectin treated eggs at three concentrations; (A) 5 mg/mL with 70% percent of inhibition of hatching, (B) 2.5 mg/mL with 63% percent of inhibition of hatching, and (C) 0.38 mg/mL with 21.7% percent of inhibition of hatching; Figure S3: Formalin treated eggs at concentration 200 mg/mL with 34.1% percent of inhibition of hatching, viable hatched larvae; Figure S4: Carvacrol treated eggs at two concentrations; (A) 25 mg/mL with 100% percent of inhibition of hatching with collapsed eggs (B) 12.5 mg/mL with 72% percent of inhibition of hatching collapsed eggs and larvae.
