# Supplementary material for: Toxicity of Common Acaricides, Disinfectants, and Natural Compounds against Eggs of Rhipicephalus annulatus
Source: Pathogens. 2024 Sep 24;13(10):824. doi: 10.3390/pathogens13100824 (PMC11510607; doi:10.3390/pathogens13100824)
Supplement: Supplementary file 1 [file pathogens-13-00824-s001.zip › pathogens-3171004-supplementary.pdf]

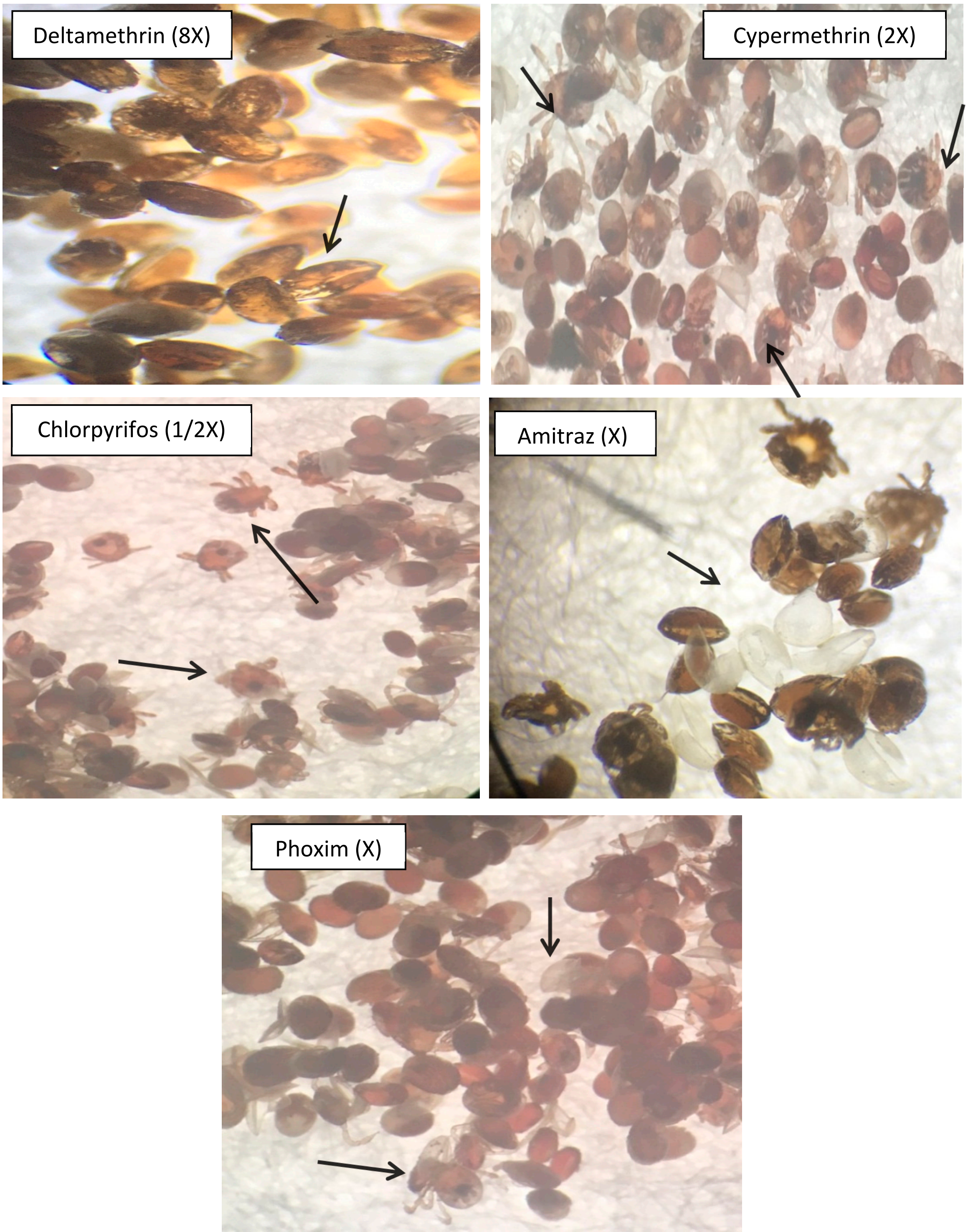

Figure S1: A. Deltamethrin treated eggs at concentration 8X with 82% percent of inhibition of hatching (IH%), with eggs collapsed and destructed eggs. B. Cypermethrin treated eggs at concentration 2X with IH% = 14.33% and large number of viable larvae. C. Chlorpyrifos treated eggs at concentration 1/2X with IH% = 16.3% and large number of viable larvae. D. Amitraz treated eggs at concentration X with IH% = 64% and shells of hatched eggs and larvae. E. Phoxim treated eggs at concentration X with IH% = 61.3% and shell of hatched eggs and larvae.

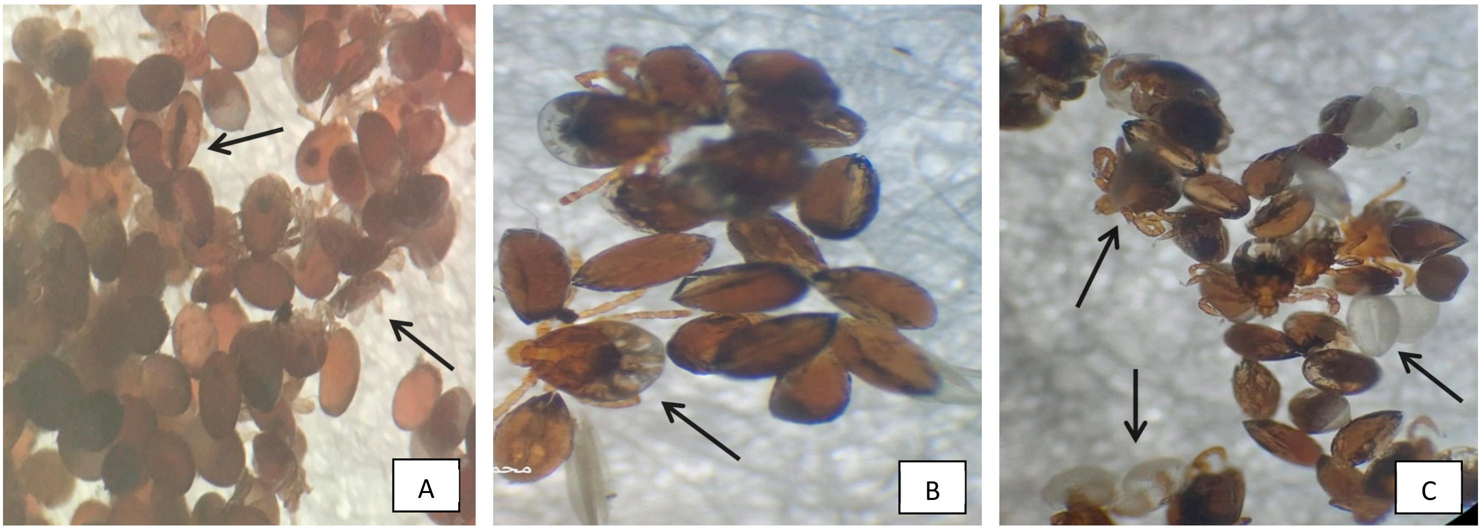

Figure S2: Ivermectin treated eggs at three concentrations; (A) 5 mg/mL with 70% percent of inhibition of hatching, (B) 2.5 mg/mL with 63 % percent of inhibition of hatching, and (C) 0.38 mg/mL with 21.7% percent of inhibition of hatching.

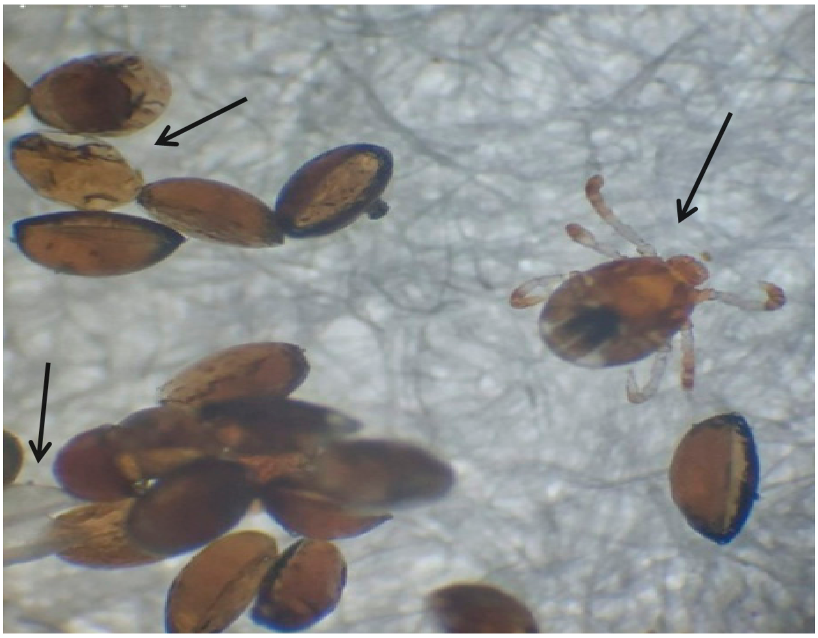

Figure S3: Formalin treated eggs at concentration 200mg/mL with 34.1% percent of inhibition of hatching, viable hatched larvae.

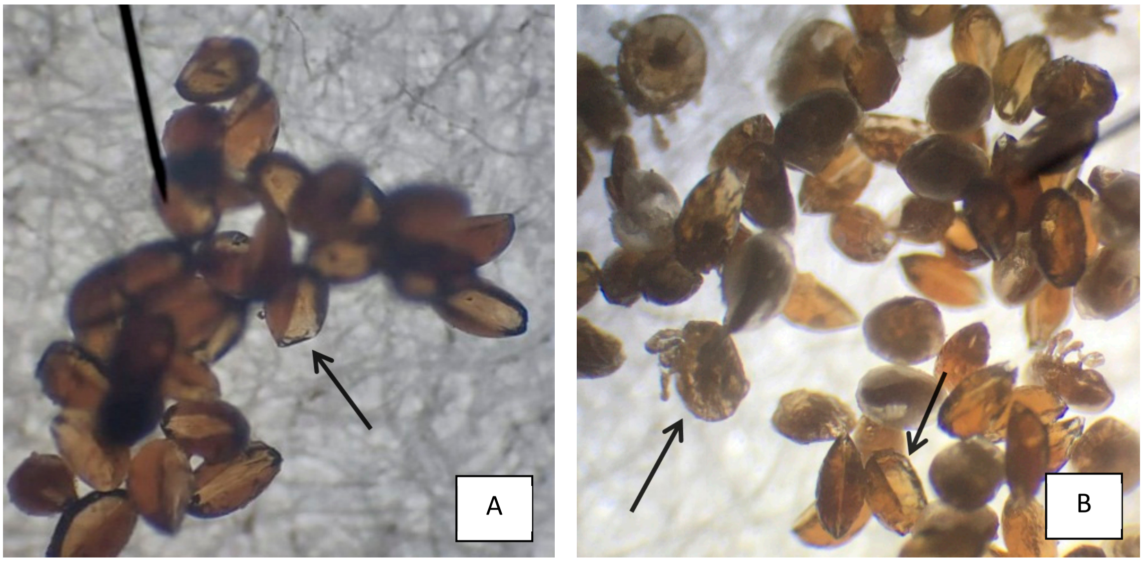

Figure S4: Carvacrol treated eggs at two concentrations; (A) 25 mg/mL with 100% percent of inhibition of hatching with collapsed eggs (B) 12.5mg/mL with 72 % percent of inhibition of hatching collapsed eggs and larvae.
